# Evolution of Social Predictive Brains?

**DOI:** 10.3389/fpsyg.2012.00414

**Published:** 2012-10-17

**Authors:** Elliot Clayton Brown, Martin Brüne

**Affiliations:** ^1^Research Department of Cognitive Neuropsychiatry and Preventative Medicine, LWL University Hospital BochumBochum, Germany; ^2^International Graduate School of Neuroscience, Ruhr University BochumBochum, Germany

Clark’s (in press) paper gives us an adept introduction into the potential for the concept of the predictive brain to provide us with an integrative and unifying framework to explain cognition. Indeed, this raises an abundance of new questions for possible future work to address. It also highlights the necessity for much gap-filling that will arise through the inevitable application of these principles to different aspects of cognition, as the breadth of the promise of the predictive brain in explaining neural and psychological phenomena becomes wider. Clark touches on some possible expansions of the predictive brain into the realm of social neuroscience. In this commentary, the relatively new hypothesis of the “social predictive brain” is presented. We argue that there is already a large body of empirical and theoretical neuroscientific work from neurophysiological, behavioral, and computational perspectives that provide substantial evidence for a fundamental role for predictive mechanisms in the processing of social information and in social interaction. Further to this, we propose that the evolution of social cognitive processes, or in other words, the evolution of the social brain, has been built upon the increasing complexity of the predictive mechanisms of perception, action, and learning, which Clark outlines in his paper.

To successfully interact with others, we must be able to interpret and understand others’ behavior, and this often involves predicting their cognitive and emotional mental states. It is likely that this is largely formed from our own perceptual and motor experiences and facilitated by the expectations acquired from previous social experience. In recent years, more empirical work has confirmed the existence of shared neural representations in social interaction, in that similar patterns of neural activity are recruited during one’s own experience and when observing others. In particular, the processing involved in the observation of others’ actions (Gallese et al., 1996), rewards (Ma et al., 2011), errors (van Schie et al., 2004), pain (Cheng et al., 2008), and fear (Mineka and Cook, 1993) is conceived through predictive mechanisms in both first-person and third-person experience. It is thought that these shared neural representations may form the neural basis for understanding the mental states of others. Predictive coding models of the mirror neuron system (Kilner et al., 2007) and the MOSAIC model of social interaction (Wolpert et al., 2003) are pertinent examples of the central role of predictive mechanisms and Bayesian inference in social interactive processes. Behrens et al. (2008) proposes the presence of a “social prediction error” during social valuation, and Heyes (2011) compellingly argues that social learning involves the same mechanisms as non-social learning, which may also apply to social decision-making (e.g., Yoshida et al., 2010). Expectancy violations in social contexts may be represented by a similar prediction error as that coding for the discrepancy between one’s own expectation and experience, such as in social deviance and the breaking of social norms (Harris and Fiske, 2010; Kim et al., 2011). Other evidence that suggests a fundamental role for predictive mechanisms in social interactive cognitive processes comes from work demonstrating a direct modulatory effect of social context and social information on low level perceptual and sensorimotor processing. For example, when participants were engaged in a social interaction, this improved visual discrimination and detection of biological motion (Neri et al., 2006; Manera et al., 2011). This “inverse relationship” between bottom-up perceptual input and top-down social information implies the presence of “social” forward (internally generated) models acting as top-down priors, which may be competing with other internal models for overall control, comparable to forward models of action (Wolpert and Miall, 1996).

In accordance with this body of work, it would be legitimate to argue that either the evolution of at least some social cognitive processes such as “theory of mind” emerged through “cooptation” of predictive mechanisms or vice versa, as selection pressures on sophisticated abilities to predict and manipulate the behavior of con-specifics increased in early hominid environments that became more heavily reliant on social cooperation. The modulation of predictive mechanisms by social context could be explained by a prioritized attentional orientation toward social information (Driver et al., 1999), which would likely have had some adaptive evolutionary benefits. The increasing complexity of the social environment in primates and humans may have consequently had an impact on the development of fundamental predictive mechanisms, and therefore recruiting previously non-social cognitions and neural structures for social predictive functions. This notion could also be supported by the substantial overlap seen between brain areas implicated in the “predictive brain” (Bubic et al., 2010) and the “social brain” (Abu-Akel and Shamay-Tsoory, 2011). These suggestions are compatible with the social brain hypothesis (Brothers, 1990; Dunbar, 1998). Indeed, Sallet et al. (2011) have recently shown that social network size and social status is correlated with gray matter volume in the superior temporal cortex and rostral prefrontal cortex in macaques. As an alternative account, which is at least equally complimentary to our proposal of the predictive social brain (Brown and Brüne, 2012), Barton (2012) convincingly demonstrates the benefit of using cerebellar neuron number as a more appropriate index of cognitive evolution, and consequently the evolution of social intelligence, as opposed to the more commonly used index of neocortex volume and neuron number. He also points to the work identifying the cerebellum as the source of predictive internal (forward) models of action (Wolpert et al., 1998), which have been extended to action understanding and action prediction in social interaction (Wolpert et al., 2003). One hypothesis of the evolution of the predictive social brain would be that the brain areas involved in predictive processing of social information are also shaped by the complexity of the social environment.

As the validity of the Bayesian/predictive brain hypothesis further gets put to the test in different realms of cognitive science, we will begin to reveal the ability to generalize these principles to other cognitive and psychological phenomena. However, it may be the case that we have already made substantial headway in confirming the potential breadth of applying such principles, at least in social neuroscience (Brown and Brüne, 2012). Therefore, it would be of value to take stock of what existing evidence from social neuroscience lends compatibility with the concept of the predictive brain, and how the implications from this work might impact on current standpoints on the evolution of the social brain.
